# Crystal structure of propane-1,3-diaminium squarate dihydrate

**DOI:** 10.1107/S2056989024008235

**Published:** 2024-08-30

**Authors:** Rüdiger W. Seidel, Tsonko M. Kolev

**Affiliations:** a Institut für Pharmazie, Martin-Luther-Universität Halle-Wittenberg, Wolfgang-Langenbeck-Str. 4, 06120 Halle (Saale), Germany; b Institute of Molecular Biology "Roumen Tsanev", Bulgarian Academy of Sciences, Acad. G. Bonchev-Str. Bl. 21, Sofia 1113, Bulgaria; University of Kentucky, USA

**Keywords:** propane-1,3-di­amine, squaric acid, proton-transfer compound, hydrogen bonding, polar structure, crystal structure

## Abstract

Propane-1,3-diaminium squarate dihydrate, C_3_H_12_N_2_^2+^·C_4_O_4_^2−^·2H_2_O, results from the proton-transfer reaction of propane-1,3-di­amine with squaric acid and subsequent crystallization from aqueous medium. The title compound crystallizes in the tetra­gonal crystal system (space group *P*4*bm*) with *Z* = 2. The squarate dianion belongs to the point group *D*_4*h*_ and contains a crystallographic fourfold axis. The propane-1,3-diaminium dication exhibits a *C*_2*v*_-symmetric all-*anti* conformation and resides on a special position with *mm*2 site symmetry. The orientation of the propane-1,3-diaminium ions makes the crystal structure polar in the *c-*axis direction. The solid-state supra­molecular structure features a triperiodic network of strong hydrogen bonds of the N—H⋯O and O—H⋯O types.

## Abstract 2

The crystal structure of propane-1,3-diaminium squarate dihydrate (space group *P*4*bm*, *Z* = 2) features a triperiodic N—H⋯O and O—H⋯O hydrogen-bond network with a polar *c* axis.

## 1. Chemical context

Squaric acid (H_2_C_4_O_4_; systematic name: 3,4-di­hydroxy­cyclo­but-3-ene-1,2-dione) and its derivatives have been widely studied in organic chemistry and materials science (Grus *et al.*, 2021 ▸; Laramie *et al.*, 2017 ▸; Wurm & Klok, 2013 ▸; Xia & Wang, 2017 ▸). Squaric acid analogues have also attracted attention in medicinal chemistry (Chasák *et al.*, 2021 ▸; Ruseva *et al.*, 2022 ▸). In structural chemistry, the inter­est in squaric acid and its mono- and dianions arises mainly from their planar, sym­metrical and strained mol­ecular structures and their diverse hydrogen-bonding patterns in the solid state (Allen *et al.*, 2013 ▸; Gilli *et al.*, 2001 ▸). As a strong diprotic organic acid with p*K*_a1_ = 0.59 ± 0.09 and p*K*_a2_ = 3.48 ± 0.023 at 298 K (as determined by potentiometric titrations; Schwartz & Howard, 1970 ▸), squaric acid readily forms proton-transfer compounds with nitro­gen bases and a wide variety of structurally characterized examples can be found in the Cambridge Structural Database (CSD; Groom *et al.*, 2016 ▸). In the present contribution, we describe the crystal structure of the dihydrate of the proton-transfer compound propane-1,3-diaminium squar­ate.
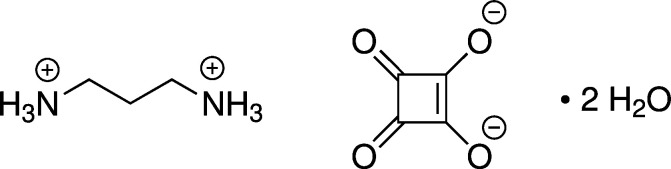


## 2. Structural commentary

**Figure 1:**
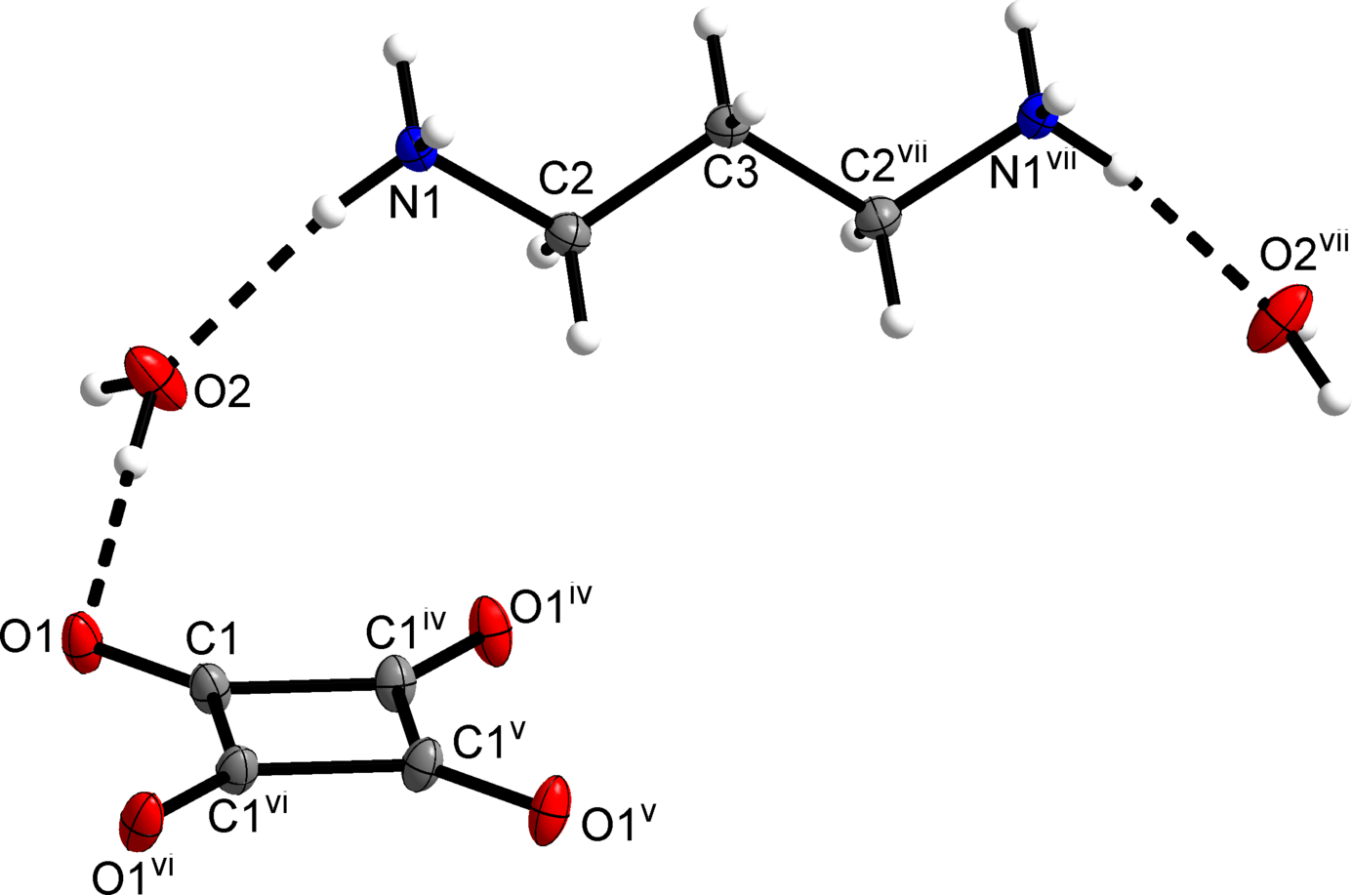
Displacement ellipsoid plot (50% probability level) of the title compound. Hydrogen atoms are shown by small spheres of arbitrary radius. Dashed lines represent hydrogen bonds. Symmetry codes: (iv) y, −x, z; (v) −x, −y, z; (vi) −y, x, z; (vii) −x + 1, −y, z;

Fig. 1 ▸ shows the mol­ecular structures of the components of the title compound in the crystal. The squarate dianion exhibits *D*_4*h*_ point-group symmetry and contains a crystallographic fourfold rotation axis with the direction [001]. The propane-1,3-diaminium dication adopts a *C*_2*v*_-symmetric all-*anti* conformation and is located on a special position with *mm*2 site symmetry in the crystal structure. The overall orientation of the mol­ecular dications renders the crystal structure polar in the *c*-axis direction. The water mol­ecule of crystallization sits on a crystallographic mirror plane perpendicular to the [110] direction.

## 3. Supra­molecular features

**Figure 2:**
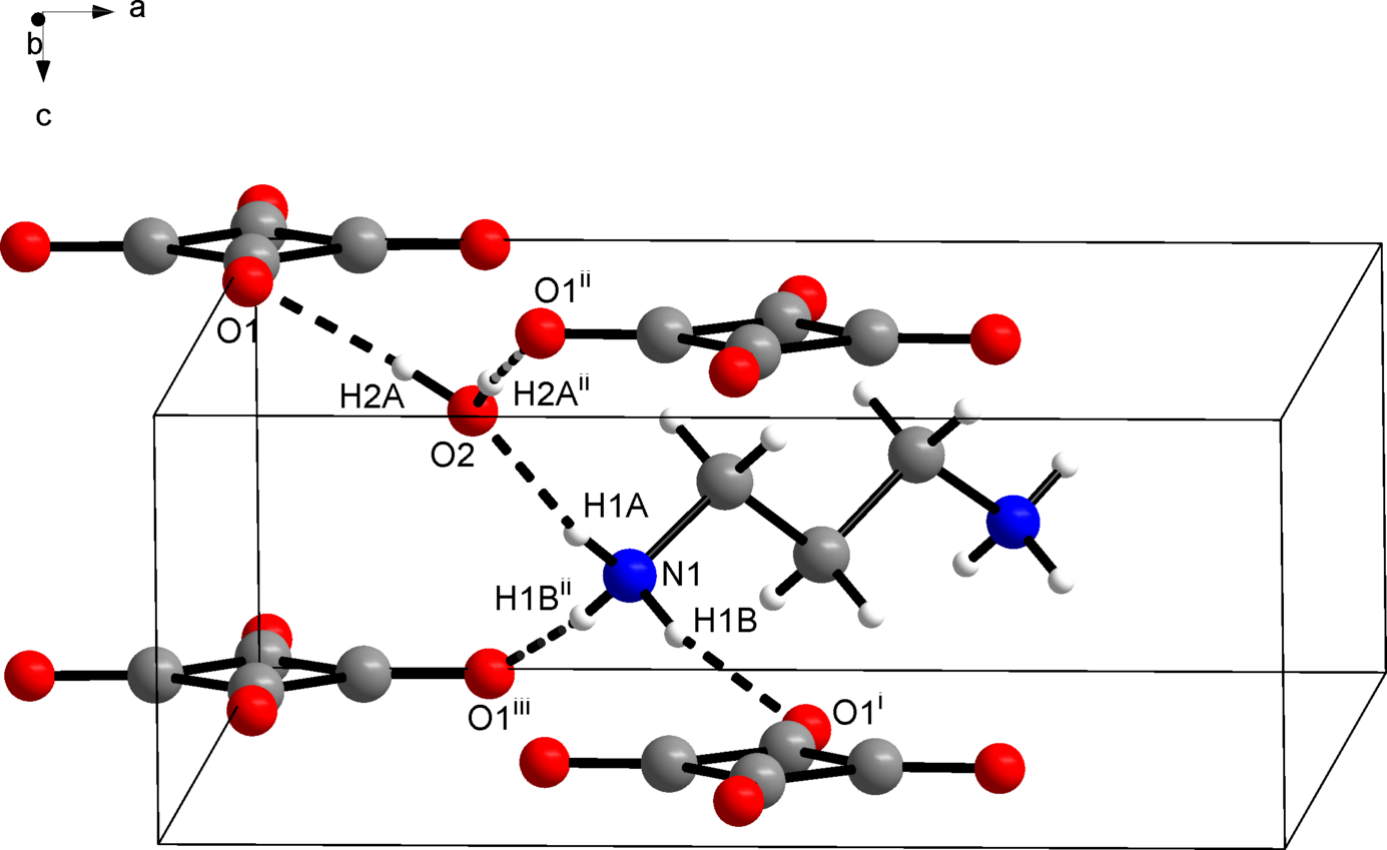
Section of the crystal structure of the title compound, viewed approximately along the [010] direction. Dashed lines illustrate hydrogen bonds. Symmetry codes: (i) *x* + 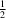, −*y* + 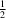, *z* + 1; (ii) −*y* + 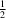, −*x* + 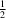, *z*; (iii) *y*, −*x*, *z* + 1.

**Figure 3:**
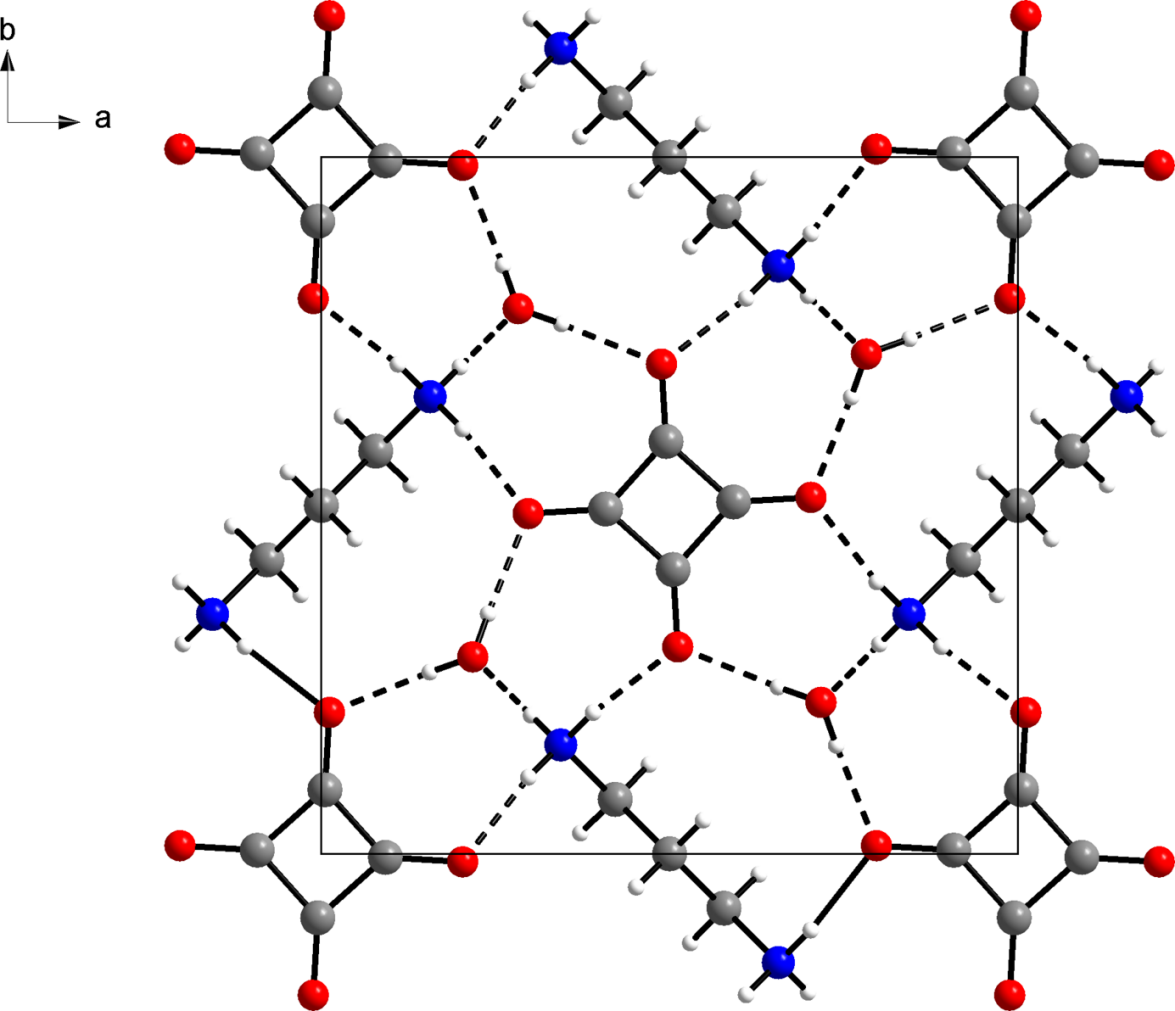
The tetra­gonal unit cell of the title compound, projected along the c-axis direction. Dashed lines illustrate hydrogen bonds. Colour scheme: C, grey; H, white; N, blue; O, red.

Apart from ion–ion inter­actions between propane-1,3-diamin­ium dications and squarate dianions, hydrogen bonding dominates the solid-state structure of the title compound. The protonated amino group joins two squarate ions *via* N—H⋯O hydrogen bonds. The remaining hydrogen-bond donor site of the aminium group forms an N—H⋯O hydrogen bond to a water mol­ecule (Fig. 2 ▸). The water mol­ecule in turn acts as hydrogen-bond acceptor towards two squarate ions, which results in a triperiodic hydrogen-bond network (Fig. 3 ▸). Table 1 ▸ lists the corresponding hydrogen-bond parameters, which are characteristic of strong hydrogen bonds (Thakuria *et al.*, 2017 ▸). The centroid–centroid distance between the squarate ions in the [001] direction corresponds to the *c* lattice parameter. A packing index of 67.8% (Kitajgorodskij, 1973 ▸), as calculated with *PLATON* (Spek, 2020 ▸), indicates a relatively open structure. This lends support to the view that strong hydrogen bonding governs the structure rather than van der Waals close packing.

## 4. Database survey

The CSD (version 5.43 with September 2022 updates; Groom *et al.*, 2016 ▸) contains >400 crystal structures with propane-1,3-diaminium cations and >100 crystal structures with squarate dianions. A structure closely related to the title compound is that of the homologous pentane-1,5-diaminium squarate dihydrate (CSD refcode: JARGAN; Ivanova & Spiteller, 2014 ▸). In contrast to the title compound, the crystal structure of JARGAN is centrosymmetric, although the pentane-1,5-diaminium cation likewise exhibits a polar (approximately) *C*_2*v*_-symmetric all-*anti* conformation. A solvent-free crystal structure of propane-1,3-diaminum bis­(hydrogen squarate) has also been published (TURQEC; Mathew *et al.*, 2002 ▸). The propane-1,3-diaminum cations in TURQEC similarly adopt an all-*anti* conformation with approximate *C*_2*v*_ point-group symmetry, but the crystal structure is centrosymmetric. Worthy of note, a low-temperature crystal structure determination of the parent free-base propane-1,3-di­amine, which is liquid at room temperature, has also been disclosed (QATVUC; Thalladi *et al.*, 2000 ▸).

## 5. Synthesis and crystallization

Starting materials were obtained from commercial sources and used as received. A solution of propane-1,3-di­amine (148 mg, 2 mmol) in 25 mL of ethanol was mixed with a solution of squaric acid (228 mg, 2 mmol) in 40 mL of distilled water. After stirring at 333 K for 4 h, the mixture was left at ambient conditions. After three weeks, colourless crystalline material was collected by filtration and air-dried. Colourless crystals of the title compound suitable for single-crystal X-ray diffraction were grown from methanol/water (1:1) by the slow evaporation method.

## 6. Refinement

Crystal data, data collection and structure refinement details are summarized in Table 2 ▸. Hydrogen-atom positions were refined freely, and *U*_iso_(H) values were set 1.2*U*_eq_(C, N, O) to improve the data/parameter ratio. The direction of the polar axis was chosen to give a Flack *x* parameter, as calculated by Parsons’ quotient method (Parsons *et al.*, 2013 ▸), close to zero. In the absence of significant anomalous scattering, however, the polar axis direction could not be determined reliably in view of the high standard uncertainty of the Flack *x* parameter (Flack & Bernardinelli, 1999 ▸). For this reason, the presence of inversion twinning also cannot be excluded.

## Supplementary Material

Crystal structure: contains datablock(s) I, global. DOI: 10.1107/S2056989024008235/pk2708sup1.cif

Structure factors: contains datablock(s) I. DOI: 10.1107/S2056989024008235/pk2708Isup2.hkl

Supporting information file. DOI: 10.1107/S2056989024008235/pk2708Isup3.cdx

Supporting information file. DOI: 10.1107/S2056989024008235/pk2708Isup4.cml

CCDC reference: 2378952

Additional supporting information:  crystallographic information; 3D view; checkCIF report

## Figures and Tables

**Table 1:** Hydrogen-bond geometry (Å, °)

*D*—H⋯*A*	*D*—H	H⋯*A*	*D*⋯*A*	*D*—H⋯*A*
N1—H1*A*⋯O2	0.85 (3)	1.92 (3)	2.758 (2)	171 (3)
N1—H1*B*⋯O1^i^	0.896 (19)	1.905 (18)	2.7887 (12)	168.4 (17)
O2—H2*A*⋯O1	0.84 (2)	1.91 (2)	2.7413 (13)	175 (2)

Symmetry code: (i) 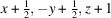.

**Table 2:** Experimental details

Crystal data	
Chemical formula	C_3_H_12_N_2_^2+^·C_4_O_4_^2−^·2H_2_O
*M*_r_	224.22
Crystal system, space group	Tetragonal, *P*4*bm*
Temperature (K)	105
*a*, *c* (Å)	11.2716 (2), 4.3310 (1)
*V* (Å^3^)	550.25 (2)
*Z*	2
Radiation type	Mo *K*α
μ (mm^−1^)	0.12
Crystal size (mm)	0.23 × 0.16 × 0.11

Data collection	
Diffractometer	Oxford Diffraction Xcalibur2
Absorption correction	Multi-scan [ABSPACK in *CrysAlis PRO* (Rigaku OD, 2023 ▸)]
*T*_min_, *T*_max_	0.928, 1.000
No. of measured, independent and observed [*I* > 2σ(*I*)] reflections	11463, 745, 721
*R*_int_	0.027
(sin θ/λ)_max_ (Å^−1^)	0.679

Refinement	
*R*[*F*^2^ > 2σ(*F*^2^)], *wR*(*F*^2^), *S*	0.027, 0.070, 1.09
No. of reflections	745
No. of parameters	54
No. of restraints	1
H-atom treatment	Only H-atom coordinates refined
Δρ_max_, Δρ_min_ (e Å^−3^)	0.38, −0.13
Absolute structure	Flack *x* determined using 298 quotients [(*I*^+^)−(*I*^−^)]/[(*I*^+^)+(*I*^−^)] (Parsons *et al.*, 2013 ▸)
Absolute structure parameter	0.1 (5)

Computer programs: *CrysAlis system* (Oxford Diffraction, 2009 ▸), *CrysAlis PRO* (Rigaku OD, 2023 ▸), *SHELXS97* (Sheldrick, 2008 ▸), *SHELXL2019/3* (Sheldrick, 2015 ▸), *DIAMOND* (Brandenburg, 2018 ▸) and *publCIF* (Westrip, 2010 ▸).

## supplementary crystallographic information

### Propane-1,3-diaminium 3,4-dioxocyclobutene-1,2-diolate dihydrate Crystal data

**Table:**

C_3_H_12_N_2_^2+^·C_4_O_4_^2^^−^·2H_2_O	*D*_x_ = 1.353 Mg m^−^^3^
*M**_r_* = 224.22	Mo *K*α radiation, λ = 0.71073 Å
Tetragonal, *P*4*b**m*	Cell parameters from 5992 reflections
*a* = 11.2716 (2) Å	θ = 3.6–28.7°
*c* = 4.3310 (1) Å	µ = 0.12 mm^−^^1^
*V* = 550.25 (2) Å^3^	*T* = 105 K
*Z* = 2	Block, colourless
*F*(000) = 240	0.23 × 0.16 × 0.11 mm

### Propane-1,3-diaminium 3,4-dioxocyclobutene-1,2-diolate dihydrate Data collection

**Table:**

Oxford Diffraction Xcalibur2 diffractometer	745 independent reflections
Radiation source: fine-focus sealed X-ray tube, Enhance (Mo) X-ray Source	721 reflections with *I* > 2σ(*I*)
Detector resolution: 8.4171 pixels mm^-1^	*R*_int_ = 0.027
ω scans	θ_max_ = 28.9°, θ_min_ = 3.6°
Absorption correction: multi-scan [ABSPACK in CrysAlisPro (Rigaku OD, 2023)]	*h* = −15→15
*T*_min_ = 0.928, *T*_max_ = 1.000	*k* = −15→14
11463 measured reflections	*l* = −5→5

### Propane-1,3-diaminium 3,4-dioxocyclobutene-1,2-diolate dihydrate Refinement

**Table:**

Refinement on *F*^2^	Secondary atom site location: difference Fourier map
Least-squares matrix: full	Hydrogen site location: difference Fourier map
*R*[*F*^2^ > 2σ(*F*^2^)] = 0.027	Only H-atom coordinates refined
*wR*(*F*^2^) = 0.070	*w* = 1/[σ^2^(*F*_o_^2^) + (0.045*P*)^2^ + 0.0625*P*] where *P* = (*F*_o_^2^ + 2*F*_c_^2^)/3
*S* = 1.09	(Δ/σ)_max_ < 0.001
745 reflections	Δρ_max_ = 0.38 e Å^−^^3^
54 parameters	Δρ_min_ = −0.13 e Å^−^^3^
1 restraint	Absolute structure: Flack *x* determined using 298 quotients [(*I*^+^)-(*I*^-^)]/[(*I*^+^)+(*I*^-^)] (Parsons *et al.*, 2013)
Primary atom site location: structure-invariant direct methods	Absolute structure parameter: 0.1 (5)

### Propane-1,3-diaminium 3,4-dioxocyclobutene-1,2-diolate dihydrate Special details

**Table:**

Geometry. All esds (except the esd in the dihedral angle between two l.s. planes) are estimated using the full covariance matrix. The cell esds are taken into account individually in the estimation of esds in distances, angles and torsion angles; correlations between esds in cell parameters are only used when they are defined by crystal symmetry. An approximate (isotropic) treatment of cell esds is used for estimating esds involving l.s. planes.

### Propane-1,3-diaminium 3,4-dioxocyclobutene-1,2-diolate dihydrate Fractional atomic coordinates and isotropic or equivalent isotropic displacement parameters (Å^2^)

**Table:**

	*x*	*y*	*z*	*U*_iso_*/*U*_eq_	
C1	0.00512 (11)	0.09200 (10)	0.0161 (4)	0.0143 (3)	
C2	0.42195 (10)	0.07805 (10)	0.5294 (5)	0.0137 (4)	
H2	0.4703 (15)	0.1281 (16)	0.407 (5)	0.016*	
C3	0.500000	0.000000	0.7310 (7)	0.0129 (5)	
H3	0.5480 (17)	0.0480 (17)	0.851 (6)	0.015*	
N1	0.34377 (9)	0.15623 (9)	0.7173 (4)	0.0126 (3)	
H1A	0.3011 (18)	0.1989 (18)	0.601 (7)	0.015*	
H1B	0.3902 (15)	0.2026 (15)	0.834 (5)	0.015*	
O1	0.01160 (8)	0.20294 (8)	0.0165 (3)	0.0188 (3)	
O2	0.21736 (10)	0.28264 (10)	0.2827 (4)	0.0244 (4)	
H2A	0.1556 (18)	0.2608 (18)	0.191 (5)	0.029*	

### Propane-1,3-diaminium 3,4-dioxocyclobutene-1,2-diolate dihydrate Atomic displacement parameters (Å^2^)

**Table:**

	*U*^11^	*U*^22^	*U*^33^	*U*^12^	*U*^13^	*U*^23^
C1	0.0114 (5)	0.0119 (6)	0.0196 (5)	0.0002 (4)	−0.0025 (5)	0.0017 (7)
C2	0.0144 (5)	0.0144 (5)	0.0123 (7)	0.0006 (6)	−0.0005 (6)	0.0005 (6)
C3	0.0132 (7)	0.0132 (7)	0.0124 (11)	0.0005 (9)	0.000	0.000
N1	0.0117 (5)	0.0117 (5)	0.0144 (6)	0.0004 (5)	−0.0013 (5)	0.0013 (5)
O1	0.0149 (4)	0.0098 (4)	0.0316 (5)	−0.0004 (3)	−0.0064 (5)	0.0014 (5)
O2	0.0197 (5)	0.0197 (5)	0.0337 (9)	−0.0071 (6)	−0.0117 (5)	0.0117 (5)

### Propane-1,3-diaminium 3,4-dioxocyclobutene-1,2-diolate dihydrate Geometric parameters (Å, º)

**Table:**

C1—O1	1.2527 (14)	C3—H3	0.92 (3)
C1—C1^i^	1.4687 (15)	C3—H3^iv^	0.92 (3)
C1—C1^ii^	1.4687 (15)	N1—H1A	0.85 (3)
C2—N1	1.488 (2)	N1—H1B	0.896 (19)
C2—C3	1.520 (3)	N1—H1B^iii^	0.896 (19)
C2—H2	0.946 (19)	O2—H2A	0.84 (2)
C2—H2^iii^	0.946 (19)	O2—H2A^iii^	0.84 (2)
			
O1—C1—C1^i^	135.16 (13)	C2^iv^—C3—H3	108.8 (8)
O1—C1—C1^ii^	134.84 (13)	C2—C3—H3^iv^	108.8 (8)
C1^i^—C1—C1^ii^	89.999 (1)	C2^iv^—C3—H3^iv^	108.8 (8)
N1—C2—C3	111.80 (18)	H3—C3—H3^iv^	112 (3)
N1—C2—H2	107.1 (11)	C2—N1—H1A	110 (2)
C3—C2—H2	109.4 (11)	C2—N1—H1B	108.0 (11)
N1—C2—H2^iii^	107.1 (11)	H1A—N1—H1B	109.7 (14)
C3—C2—H2^iii^	109.4 (11)	C2—N1—H1B^iii^	108.0 (11)
H2—C2—H2^iii^	112 (2)	H1A—N1—H1B^iii^	109.7 (14)
C2—C3—C2^iv^	109.9 (2)	H1B—N1—H1B^iii^	111 (2)
C2—C3—H3	108.8 (8)	H2A—O2—H2A^iii^	105 (3)
			
N1—C2—C3—C2^iv^	180.000 (1)		

Symmetry codes: (i) −*y*, *x*, *z*; (ii) *y*, −*x*, *z*; (iii) −*y*+1/2, −*x*+1/2, *z*; (iv) −*x*+1, −*y*, *z*.

### Propane-1,3-diaminium 3,4-dioxocyclobutene-1,2-diolate dihydrate Hydrogen-bond geometry (Å, º)

**Table:**

*D*—H···*A*	*D*—H	H···*A*	*D*···*A*	*D*—H···*A*
C2—H2···O1^v^	0.946 (19)	2.590 (19)	3.4712 (18)	155.1 (14)
N1—H1*A*···O2	0.85 (3)	1.92 (3)	2.758 (2)	171 (3)
N1—H1*B*···O1^vi^	0.896 (19)	1.905 (18)	2.7887 (12)	168.4 (17)
O2—H2*A*···O1	0.84 (2)	1.91 (2)	2.7413 (13)	175 (2)

Symmetry codes: (v) *x*+1/2, −*y*+1/2, *z*; (vi) *x*+1/2, −*y*+1/2, *z*+1.
